# In Vitro Bioassay for Damage-Associated Molecular Patterns Arising from Injured Oral Cells

**DOI:** 10.3390/bioengineering11070687

**Published:** 2024-07-05

**Authors:** Layla Panahipour, Chiara Micucci, Benedetta Gelmetti, Reinhard Gruber

**Affiliations:** 1 Department of Oral Biology, University Clinic of Dentistry, Medical University of Vienna, 1090 Vienna, Austria; layla.panahipour@meduniwien.ac.at (L.P.); chiara.micucci@studenti.unipr.it (C.M.); benedetta.gelmetti@studenti.unipr.it (B.G.); 2 Department of Periodontology, School of Dental Medicine, University of Bern, 3010 Bern, Switzerland; 3 Austrian Cluster for Tissue Regeneration, 1200 Vienna, Austria

**Keywords:** DAMPs, injured oral epithelial cells, periodontitis, alarmins, necrosis, gingival fibroblast, oral squamous carcinoma cells, STC1, AREG, C11orf96

## Abstract

Gingival fibroblasts are a significant source of paracrine signals required to maintain periodontal homeostasis and to mediate pathological events linked to periodontitis and oral squamous cell carcinomas. Among the potential paracrine signals are stanniocalcin-1 (STC1), involved in oxidative stress and cellular survival; amphiregulin (AREG), a growth factor that mediates the cross-talk between immune cells and epithelial cells; chromosome 11 open reading frame 96 (C11orf96) with an unclear biologic function; and the inflammation-associated prostaglandin E synthase (PTGES). Gingival fibroblasts increasingly express these genes in response to bone allografts containing remnants of injured cells. Thus, the gene expression might be caused by the local release of damage-associated molecular patterns arising from injured cells. The aim of this study is consequently to use the established gene panel as a bioassay to measure the damage-associated activity of oral cell lysates. To this aim, we have exposed gingival fibroblasts to lysates prepared from the squamous carcinoma cell lines TR146 and HSC2, oral epithelial cells, and gingival fibroblasts. We report here that all lysates significantly increased the transcription of the entire gene panel, supported for STC1 at the protein level. Blocking TGF-β receptor 1 kinase with SB431542 only partially reduced the forced expression of STC1, AREG, and C11orf96. SB431542 even increased the PTGES expression. Together, these findings suggest that the damage signals originating from oral cells can change the paracrine activity of gingival fibroblasts. Moreover, the expression panel of genes can serve as a bioassay for testing the biocompatibility of materials for oral application.

## 1. Introduction

The oral cavity, with its mucosa, dentition, and tooth-supporting periodontal structures, is unique because it is permanently exposed to masticatory forces and microbial burden [1,2,3]. Consequently, the oral tissues undergo permanent self-renewal to maintain tissue homeostasis by responding to local tissue damage [2,3]. Damage or a severe infection causes the local release of molecules from necrotic damaged or dying cells, signalling the need for repair to other vital cells, in an autocrine and paracrine mode of action. These signalling molecules, the damage-associated molecular patterns (DAMPs), also termed danger signals or alarmins, are released from damaged or dying cells and typically initiate an innate immune response. The molecular nature and classification, the cellular origin and sensing of DAMPs, and the clinical therapeutic strategies targeting DAMPs in human diseases are summarized by fundamental reviews related to DAMPs in inflammation and diseases [4,5]. Thus, necrosis, as defined by an irreversible cell injury due to pathological processes that cause plasma membrane rupture, allows the release of DAMPs that, in turn, trigger a local cellular response. Necrosis occurs fast and is not regulated on a molecular level as it occurs in response to a severe chemical or physical impact. In vitro, we have established sonication to prepare cell lysates, which is a destructive physical impact. In vivo, necrosis can be linked to invasive dental treatments such as scaling and root planing, as well as surgical procedures [6], bone drilling [7,8], cryosurgery [9], and piezosurgery [10]. Moreover, the histopathological significance of necrosis in oral lesions, including odontogenic cysts and tumors, salivary gland pathology, and epithelial malignancies, has gained increasing attention [11]. There is thus fundamental interest in better understanding the cellular and molecular consequences of oral cell necrosis: What happens in the localized area where necrotic oral cells release their DAMPs?

DAMPs are initially endogenous molecules that, upon cell damage, become accessible by the neighboring cells equipped with a panel of pattern recognition receptors, allowing them to respond to the DAMPs [4,5]. This process is comparable to a cell response caused by bacterial virulence factors such as LPS and flagellin, termed necroinflammation [12]. DAMPs comprise a large spectrum of molecules released from various compartments of damaged cells, such as the cytoplasm, the plasma membrane, the nucleus, the endoplasmic reticulum, and the mitochondria. Clinically, there is increasing interest in DAMPs related to tissue damage and periodontitis. For instance, uric acid has recently been linked to periodontitis [13]. Moreover, heat shock protein 70 [14], cyclophilin A [15], and amyloid beta [16] levels in the gingival crevicular fluid were expressed higher at periodontitis sites. Other examples of DAMPs found to be elevated in periodontitis are high-mobility group box-1 (HMGB1) and HMGN2, both transcription factors [17,18], and IL1 [19]. We can further propose TGF-β to be among the DAMPs as the growth factor is stored in the extracellular matrix [20] and identified in lysates of the human squamous carcinoma cell lines HSC2 and TR146, of gingival fibroblasts, but also in the supernatant of demineralized bone matrix [21]; all preparations have caused a robust increase in IL11, a TGF-β target gene, in the gingival fibroblasts cells [22,23]. Moreover, TGF-β is present in the crevicular fluid, particularly in periodontitis [24]. This list is not complete, as not all DAMPs have been studied in periodontitis.

Even though DAMPs usually cause an immune response, their impact might be more complex and should not be restricted to inflammation. Potential candidate genes that DAMPs might regulate are amphiregulin (AREG), stanniocalcin-1 (STC1), C11orf96, and prostaglandin E synthase (PTGES), as they were increasingly expressed when gingival fibroblasts are exposed to lysates of bone allografts [22]. Bone originating from human donors is cleaned, optionally demineralized and sterilized; but all the sequential processing steps cannot rule out the remaining presence of damaged cells; thus, DAMPs [25]. Support for this assumption comes from our observation that lysates prepared from IDG-SW3 osteocyte-like cells increase the respective gene panel in fibroblasts [23]. Moreover, bone allografts possess TGF-β activity, suggesting a possible role of TGF-β serving as a DAMP [21]. For instance, AREG is among the possible TGF-β-regulated molecules [26]. AREG has an anabolic function in mouse bone homeostasis [27,28]. AREG is also relevant for intestinal epithelial regeneration after radiation injury [29]. Moreover, damage-dependent activation causes thymic lymphoid cells to produce AREG, promoting epithelial cell differentiation [30]. Also, dying tumor cells create AREG-based extracellular environments [31], and bioactive lipids can increase AREG in cancer-associated fibroblasts [32]. STC1 is involved in skeletal development [33], targets macrophages [34], and protects endothelial cells from inflammatory injury [35]. The role of chromosome 11 open reading frame 96 (C11orf96) remains to be discovered, but at least we know that C11orf96, being conserved among different species, is associated with several transmembrane family and zinc finger proteins. C11orf96 is distributed in all tissues and organs [36]. In contrast, PTGES function is well understood; for instance, PTGES contributes to the pathogenesis of collagen-induced arthritis [37] and LPS-induced alveolar bone loss [38]. Thus, there is reason to assume that these candidate genes can, at least partially, mediate the response of vital fibroblasts to DAMPs released from damaged cells. 

We, therefore, hypothesize that increased expression of STC1, AREG, C11orf96, and PTGES might change the microenvironment of gingival fibroblasts and that their expression is driven by DAMPs released from the cellular remnants of damaged oral cells.

## 2. Material and Methods

### 2.1. Cell Lines

Gingival fibroblasts of human origin were derived from small gingival tissue specimens procured during wisdom teeth extraction from three healthy individuals who provided informed consent. The protocol was endorsed by the Ethical Committee of the Medical University of Vienna (EK Nr. 631/2007). Fibroblasts obtained by explant cultures exhibited the typical spindle-shaped morphology. The oral squamous cell carcinoma cell lines HSC2 and TR146 were initially acquired from the Health Science Research Resources Bank in Sennan, Japan, and from the European Collection of Authenticated Cell Cultures, respectively. These cells were cultured in DMEM supplemented with 10% foetal calf serum (FCS) and 1% antibiotics (Invitrogen Corporation, Carlsbad, CA, USA). Primary oral epithelial cells were taken from the epithelial layer of human gingiva harvested from the extracted third molars of patients who had given informed and written consent (EK NR 631/2007) and cultivated in a keratinocyte growth medium (PromoCell, Heidelberg, Germany). For the bioassay, gingival fibroblasts were seeded at 3 × 10^4^ cells /cm^2^ into 24-well plates; the cells were treated with undiluted necrotic cell lysate or growth medium for 18 h followed by RT-qPCR gene expression analysis and immunoassay. If indicated, 10 µM of the TGF-β RI kinase inhibitor SB431542 (Calbiochem, Merck, Billerica, MA, USA) or 10 ng/mL TGF-β1 (ProSpec-Tany TechnoGene Ltd., Ness-Ziona, Israel) was applied.

### 2.2. Cell Lysates 

Cells were suspended at a concentration of 4 × 10^6^ cells/mL in DMEM supplemented with antibiotics. The cell suspensions were subjected to sonication three times, each lasting 15 s (Sonoplus, Bandelin Electronic GmbH & Co. KG, Berlin, Germany). The resulting necrotic cell lysates were centrifuged at 2600 relative centrifugal force (RCF) for 5 min (Eppendorf 5420, Eppendorf SE, Hamburg, Germany). Subsequently, all supernatants, now classified as necrotic cell lysates, were freshly prepared for each independent experiment. The cell pellet was discarded. 

### 2.3. Reverse Transcription Quantitative Real-Time PCR (RT-qPCR) 

Total RNA was isolated utilizing the ExtractMe total RNA kit manufactured by Blirt S.A., Gdańsk, Poland. Subsequently, cDNA was synthesized via reverse transcription of the isolated total RNA, employing LabQ technology from Labconsulting, Vienna, Austria. PCR amplification was conducted using LabQ equipment from Labconsulting, Vienna, Austria, and performed on a CFX Connect™ Real-Time PCR Detection System manufactured by Bio-Rad Laboratories, Hercules, CA, USA). The selection of target genes was based on RNAseq of fibroblasts exposed to liquid extracts of bone allografts where AREG, LIF, IL11, GK, STC1, C11orf96 (>60-fold increase) but also PTGES (28-fold increase) were identified as strongly regulated genes [22] and supported by findings with lysates prepared from IDG-SW3 osteocyte-like cells [23]. In our recent work, we have focused on IL11, AREG, C11orf96, STC1, and GK with RT-qPCR [22]. Primer sequences and melting curves are indicated in Supplementary Materials. The quantification of individual mRNA levels was normalized to the expression of GAPDH using the ∆∆Ct method. Relative mRNA expression levels are normalized to the unstimulated control. 

### 2.4. Immunoassay

Necrotic cell lysates were applied to gingival fibroblasts for 16–18 h. The supernatants were obtained and centrifuged before being kept for three weeks at −20 °C. Per the manufacturer’s instructions, an immunoassay determined the amount of STC1 in the supernatant (DY2958, R&D Systems, Minneapolis, MN, USA).

### 2.5. Statistical Analysis

All experiments were performed at least four times. Statistical analyses were performed with ratio-paired *t*-tests. Analyses were performed using Prism v.9 (GraphPad Software; San Diego, CA, USA). Significance was set at *p* < 0.05. Data are presented as scatter blots and in the Supplementary Materials as bar graphs with mean and standard deviation.

## 3. Results

### 3.1. Necrotic Oral Cell Lysate Drives Gene Expression in Gingival Fibroblasts 

**Figure 1:**
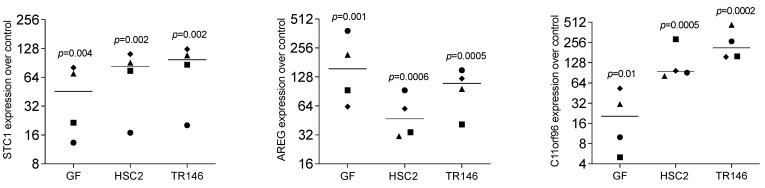
RT-PCR analysis of gingival fibroblasts in the presence of necrotic cell lysate of gingival fibroblasts, HSC2, and TR146. Data points represent four independent experiments. Data were normalized to untreated control, giving as x-fold changes. The analysis was based on a ratio-paired *t*-test.

**Figure 2:**
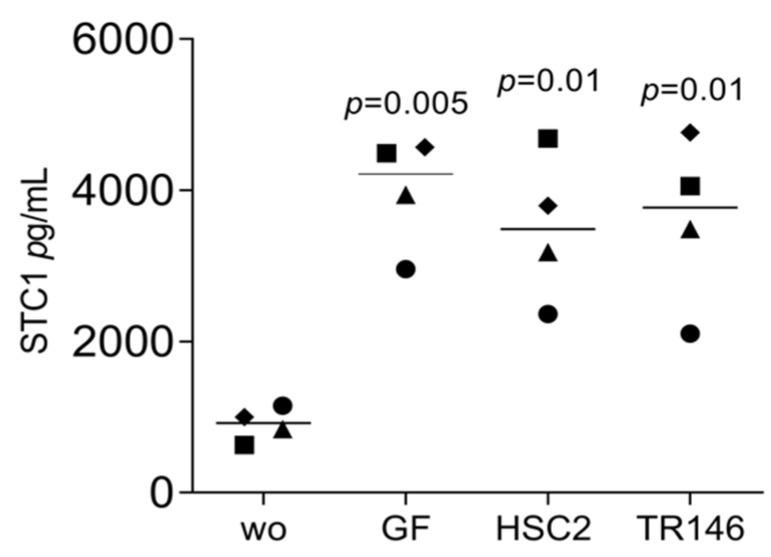
Immunoassay analysis of gingival fibroblasts in the presence of necrotic cells. Gingival fibroblasts were incubated with necrotic cell lysate overnight, and the immunoassay showed an increase in STC1. Data points represent four independent experiments. The analysis was based on a ratio-paired *t*-test.

**Figure 3:**
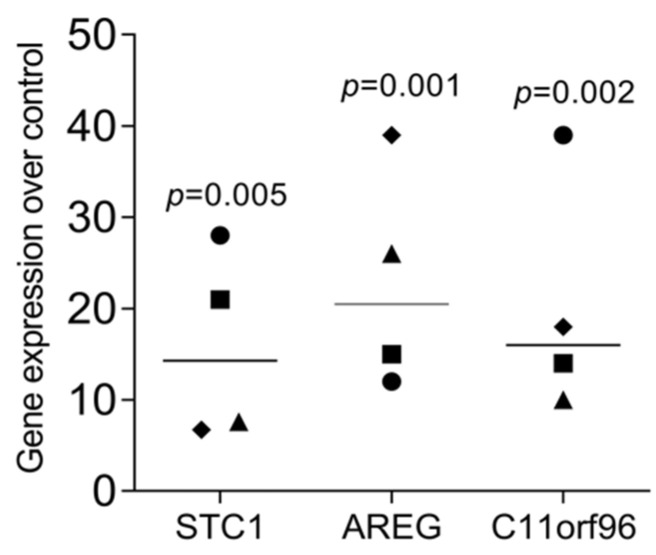
RT-PCR analysis of gingival fibroblasts in necrotic cell lysate of primary epithelial cell lysate. Gingival fibroblasts incubated with necrotic cell lysates overnight, and the gene expression analysis showed an increase in STC1, AREG, and C11orf96 in gingival fibroblasts. Data points represent four independent experiments. Data were normalized to untreated control, giving as x-fold changes. The analysis was based on a ratio-paired *t*-test.

We have recently identified a panel of genes that are increasingly expressed when gingival fibroblasts are exposed to liquid extracts of bone allografts, a preparation from human donor bone possibly containing DAMPs [22]. These DAMPs may originate from damaged cells and the allogenic bone matrix. Moreover, dental procedures are often invasive and cause cell damage; however, the local response to the DAMPs originating from damaged oral cells remains enigmatic. This clinical scenario has prompted us to simulate the harsh processing of allografts and the cell damage that might be related to invasive dental treatment by exposing dispersed cells to sonication. To rule out any toxicity, we measured the incorporation of tritium-labelled thymidine. Lysate from HSC2 (1.72 ± 0.17-fold) and TR146 (1.51 ± 0.12-fold) moderately increased DNA synthesis in gingival fibroblasts. We then showed that the respective supernatant representing the necrotic cell lysates caused gingival fibroblasts to change the expression of our allograft-sensitive genes dramatically [22]: STC1, AREG, and C11orf96 (Figure 1). While lysates from HSC2 and TR146 strongly increased the entire gene panel, lysates from gingival fibroblasts only modestly increased C11orf96 expression (Figure 1). Consistently, on the protein level, gingival fibroblasts increasingly produced STC1 when exposed to lysates from HSC2, TR146, and gingival fibroblasts (Figure 2). Also, lysates prepared from nontransformed primary oral epithelial cells could similarly provoke STC1, AREG, and C11orf96 expression on gingival fibroblasts (Figure 3). 

### 3.2. STC1, AREG, and C11orf96 Are Increased by Oral Cell Lysate Depending on SB431542

**Figure 4:**
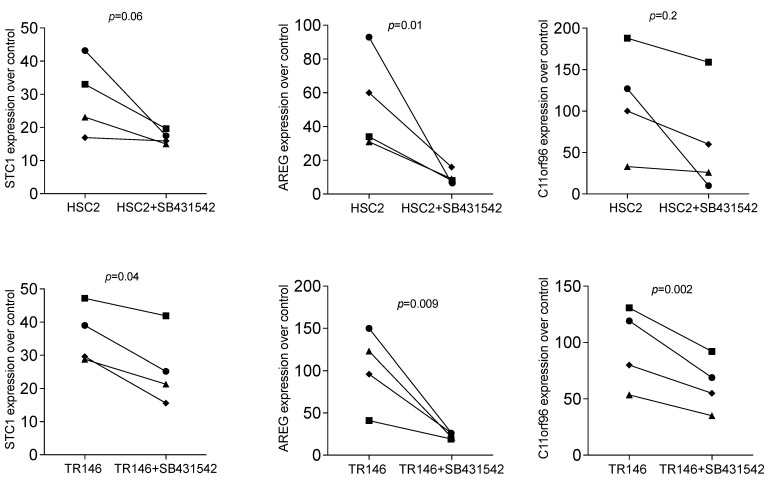
RT-PCR analysis of gingival fibroblasts incubated with HSC2 and TR146 necrotic cell lysates overnight in SB431542. RT-PCR analysis of gingival fibroblasts incubated with necrotic cell lysates with and without the TGF-β RI kinase inhibitor SB431542. Expression analysis showed that blocking TGF-β signalling reduced necrotic cell lysate-induced STC1, AREG, and C11orf96 expression in gingival fibroblasts. Data points represent four independent experiments. Data were normalized to untreated control, giving as x-fold changes. The analysis was based on a ratio-paired *t*-test.

Based on our recent bone allograft study, we introduced the TGF-β RI kinase inhibitor SB431542 to understand if all potential target genes are activated via the same signalling pathway. Previously, SB431542 reduced allograft-induced expression of AREG and C11orf96 but not of STC1 [22]. Consistent with what we have observed with bone allografts [22], SB431542 significantly reduced the HSC2 and TR146-lysate-induced expression of AREG and C11orf96; we also identified STC1 to be sensitive to SB431542 in the present study (Figure 4). 

### 3.3. PTGES Is Regulated by Oral Cell Lysate but Enhanced by SB431542

**Figure 5:**
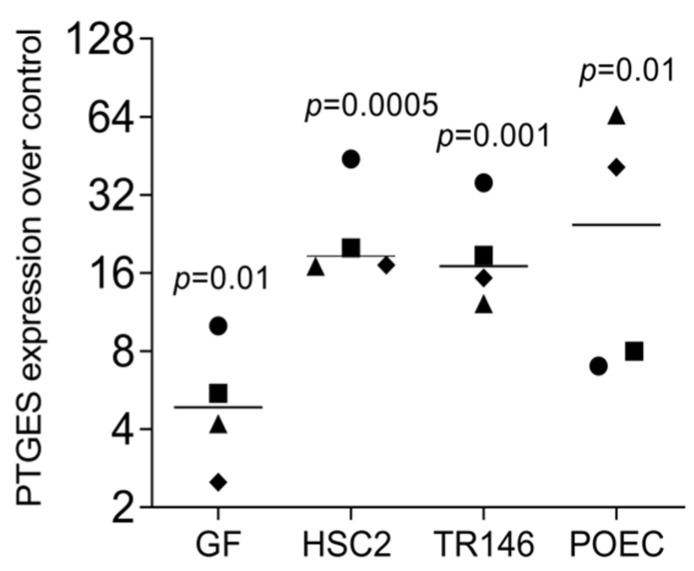
RT-PCR analysis of gingival fibroblasts incubated with gingival fibroblast (GF), HSC2, and TR146, and primary oral epithelial cell (POEC) cell lysates overnight. Gene expression analysis showed an increase in PTGES. Data points represent four independent experiments. Data were normalized to untreated control giving as x-fold changes. The analysis was based on a ratio-paired *t*-test.

**Figure 6:**
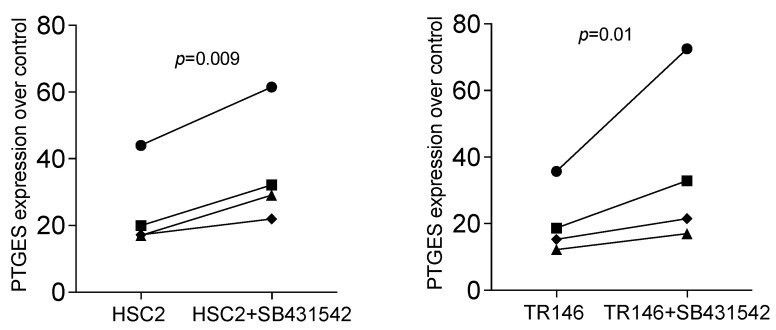
RT-PCR analysis of gingival fibroblasts incubated with HSC2 and TR146 necrotic cell lysates overnight with and without the TGF-β RI kinase inhibitor SB431542. Expression analysis showed that blocking TGF-β signalling increased necrotic cell lysate-induced expression of PTGES in gingival fibroblasts. Data points represent four independent experiments. Data were normalized to untreated control giving as x-fold changes. The analysis was based on a ratio-paired *t*-test.

**Figure 7:**
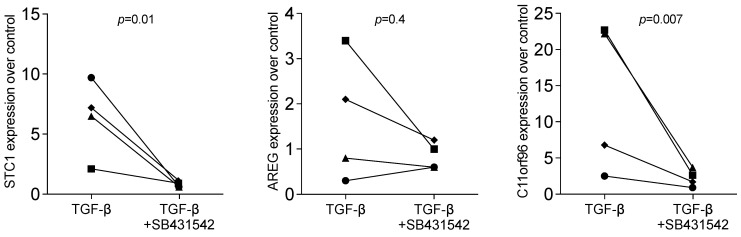
RT-PCR analysis of gingival fibroblasts incubated with recombinant TGF-β overnight. with and without the TGF-β RI kinase inhibitor SB431542. Expression analysis showed that blocking TGF-β signalling reduced induced expression in gingival fibroblasts. Data points represent four independent experiments. Data were normalized against untreated control cells with x-fold changes compared to the untreated cells. The analysis was based on a ratio-paired *t*-test.

Therefore, we returned to our allograft paper and screened the RNAseq data for strongly regulated genes that might be independently regulated by TGF-β signalling [22]. We have identified PTGES, the prostaglandin E synthase, typically involved in catabolic inflammatory conditions [37,38]. There is reason to suggest that PTGES is an additional candidate for a bioassay showing the TGF-β-independent effects, perhaps because IL1 is expressed and stored in barrier cells, in particular, dermal epithelial cells [39,40]. Indirect support for this new hypothesis comes from our observation that SB431542 failed to entirely block STC1, AREG, and C11orf96 expression, and even increased the expression of PTGES induced by HSC2 and TR146-lysate. This assumption is in line with what we have observed for cytokine expression recently [41] (Figure 5 and Figure 6). Moreover, recombinant TGF-β was less effective in driving STC1, AREG, and C11orf96 expression than the cell lysates (6.37 ± 0.21; 1.65 ± 0.25; 13.55 ± 1.04-fold change, respectively (Figure 7). Thus, other signalling pathways might be involved in how the DAMPs exert their activity on gingival fibroblasts. These preliminary findings provide the scientific basis for future research aiming to uncover the role of IL1 and other DAMPs originating from oral cells to provoke gene expression changes. 

## 4. Discussion

DAMPs are more than a technical term for damage-associated molecular patterns, which are danger signals released during tissue injury [4,5]. DAMPs originate from the body’s cells when injured [4,5] provoking a sterile inflammation by taking advantage of molecular mechanisms used to defend against bacteria and other unwanted foreign microbes. The reaction of the local healthy environment to DAMPs, particularly in the context of injured oral tissues, has only recently become a research focus, for instance, in the context of masticatory forces [1,2] and invasive procedures of drilling and implant placement [7,8]. Nevertheless, and although not extensively studied, it is reasonable to suggest that some other dental procedures are invasive as well, for instance, the use of scaling instruments to clean the root surface [6] and all kinds of surgeries [9,10], mainly when linked to temperature changes or physical forces. Apart from dental instruments, dental resins cause cell damage [42,43]. Consequently, the content of the cytoplasm and the disrupted cell membrane become DAMPs and are potentially recognized by the healthy local cells. In the oral cavity, these healthy cells include the fibroblasts of the gingiva, which are now increasingly recognized as significant sources of paracrine signals in periodontal disease [44]. Among the cells potentially suffering from tissue damage are the fibroblasts and the epithelial cells, the latter serving as a barrier towards the oral cavity. Based on this clinical scenario, we have established a bioassay where healthy gingival fibroblasts are exposed to cell lysates from injured gingival fibroblasts and epithelial cells. 

In the present study, we have not focused on the expression of inflammatory mediators such as cytokines and chemokines [41] but considered our previous observation obtained with bone allografts [22]. The allogenic human donor bone undergoes a multi-step process to remove most of the original cellular components, but of course, with cell fragments remaining in the final product [22]. Based on RNAseq analysis, we have identified a panel of genes strongly expressed by gingival fibroblasts that were exposed to an aqueous fraction of the allograft material [22] or IDG-SW3 osteocyte-like cells [23], among which were STC1, AREG, and C11orf96, as well as PTGES. The expression of AREG and C11orf96 was partially dependent on TGF-β signalling, suggesting that TGF-β originating from the allografts use this signalling pathway [22]. However, our previous study could not disclose the possible involvement of cellular components, as the effects might be caused by components of the extracellular matrix, the allograft matrix. It is thus not surprising that our previous research has led us to ask if the gene panel we have identified [22] also works as a bioassay for necrotic cell lysates, and indeed, this was the case. Impressively, lysates obtained from oral squamous cell carcinoma lines HSC2 and TR146 but also the healthy equivalent, the oral epithelial cells, all provoked the increased expression of STC1, AREG, and C11orf96 in gingival fibroblasts. The observed effects were not restricted to a specific cell type as lysates from gingival fibroblasts also provoked this gene expression. Thus, our data imply that yet-to-be-defined DAMPs, which are released from injured epithelial cells and fibroblasts, force healthy fibroblasts to express genes that purposively have autocrine/paracrine functions.

The question now arises about what a possible explanation for the gene expression changes in gingival fibroblasts is. Based on our original attempts with allografts to blame TGF-β signalling for the changes on AREG, STC1, and C11orf96 gene expression [22], we have blocked TGF-β receptor 1 kinase with SB431542. In the present study, STC1, AREG, and C11orf96 were significantly but not entirely inhibited by SB431542. Thus, the impact of the cell lysates on the expression changes cannot be explained exclusively by the activation of TGF-β signalling. In support of this notion, we have included another previously identified gene [22], PTGES, a classical inflammatory target gene, in our bioassay. As expected, SB431542 failed to decrease PTGES expression, but in contrast, SB431542 even increased HSC2- and TR146-induced PTGES expression by the fibroblasts. Thus, it can be assumed that when the potential anti-inflammatory activity of TGF-β signalling is blocked [45], pro-inflammatory DAMPs may gain predominance and further push the PTGES expression. This observation can hypothetically be explained by IL1 serving as a DAMP in epithelial cells; at least based on what is known for keratinocytes [39]. However, even though there is conistency that lysates of oral squamous carcinoma cell lines provoked a robust increase in the expression of inflammatory mediators and activate NF-kB signalling in gingival fibroblasts, the effect was independent of an IL1 receptor-associated kinase-1/4 inhibitor [41]. Nevertheless, this hypothesis will remain the scientific foundation for future research, where we plan to block other IL1 signalling pathways to better understand the inflammatory cues within the cell lysates. Future research will also focus on how dental and other biomaterial are causing the release of DAMPs from injured cells. Thus, our findings can be adapted to establish a bioassay for screening a biomaterial’s potential necrotic toxicity, simply by analysing the expression of STC1 [46], AREG [47], C11orf96 [48], and PTGES [49] in fibroblasts. At this point, we should emphasise that the quality of the expression analysis was based on sharp melting points and the use of established primer sets not limited to our research [22]. 

From a clinical perspective, and if we consider—apart from PTGES—STC1 [50], AREG [51], and perhaps also C11orf96 [36] to be autocrine/paracrine mediators, we might speculate about the potential role of this mechanism in oral wound healing and other aspects of tissue response [52,53,54]. Any potential effects of fibroblasts increasingly expressing STC1, AREG, C11orf96, and PTGES should be viewed under the premise of a temporal effect when the connective tissue is exposed to the DAMPs released from damaged cells, not to be compared to chronic inflammation. We can raise a clinical hypothesis based on in vitro studies with STC1 and AREG [44,45,46], but raising a hypothesis is challanging because the response of gingival fibroblasts to DAMPs from damaged cells is more complex than the gene panel we have identified here and previously [22,23]. Moreover, target cells other than fibroblasts should be considered in future research. It is likewise worth asking to which extent in vitro sonication represents necrotic cell damage in a clinical scenario. Equally important in this context is to study if our bioassay reflects the clinical reality where invasive dental procedures cause damaged cells to release DAMPs which in turn provoke a local response, perhaps including— but not exclusively—the expression of cytokines, STC1, AREG, etc. We can further speculate that STC1, AREG, and C11orf96 might serve as biomarkers to monitor local cell damage in a clinical setting as long as we have not identified the molecular cause, the DAMPs, responsible for changing the genetic signature of the gingival fibroblasts. 

The clinical relevance of the changing autocrine/paracrine local environment can be further discussed in the context of what is known about the single molecules; for instance, increased expression can be interpreted positively as AREG supports bone homeostasis [27,28] and regeneration of damaged epithelial cells [29,30]. Also, STC1 functions in an autocrine/paracrine manner during bone development; however, continuous overexpression impairs bone development, underlining the importance of its tight regulation [33]. Even though the potential clinical relevance of C11orf96 remains at the level of speculation, its strong regulation points towards a biological role that remains to be discovered [36]. The interpretation of increased PTGES seems easy, as prostaglandin synthesis is linked to inflammatory osteolysis in arthritis [37] and alveolar bone loss [38], but prostaglandin synthesis is also relevant for bone regeneration and homeostasis [55,56], including that of craniofacial bones [57]. Apart from research on PTGS2 knockout models, a lack of PTGES in mice caused impaired fracture healing [58]. Hence, a transient increase in PTGES caused by cell lysates could be interpreted towards support of bone regeneration. To learn more about the clinical relevance of our in vitro observations, tissue damage and recovery models using STC1, AREG, C11orf96, and PTGES knockout mice would be a feasible approach. Moreover, it would be worth identifying the expression changes in the various fibroblast populations immediately following severe tissue damage based on single-cell RNA sequencing [44]. 

In conclusion, the research presented here remains descriptive as it shows that gingival fibroblasts increasingly express STC1, AREG, C11orf96, and PTGES when exposed to cell lysates originating from damaged epithelial cells and fibroblasts. Considering these limitations, our research holds the potential to discover the underlying DAMP-related molecular mechanisms and their clinical relevance. Moreover, our findings might be the basis for toxicity screening of biomaterials in vitro. This bioassay presumably exceeds the limited borders of dentistry. The fundamental mechanisms are relevant in all kinds of biomaterial research and potentially even stretch its applicability towards the in vivo behavior of biomaterials, knowing that STC1, AREG, C11orf96, and PTGES are more than target genes of a bioassay; they possess bioactivity and might explain the local response of a tissue to a certain biomaterial. At the moment, this claim remains at the level of speculation but might inspire future research to discover the role of our target genes in experimental and clinical research.

## Data Availability

All data are available on demand.

## Supplementary Materials

The following are available online at: https://www.mdpi.com/article/10.3390/bioengineering11070687/s1, Supplementary Materials include bar graphs Figures with mean and standard deviation, the respective Tables, primer sequence and melting curves. Figure S1: RT-PCR analysis of gingival fibroblasts in the presence of necrotic cell lysate of gingival fibroblasts, HSC2, and TR146; Figure S2: Immunoassay analysis of gingival fibroblasts in the presence of necrotic cells; Figure S3: RT-PCR analysis of gingival fibroblasts in necrotic cell lysate of primary epithelial cell lysate; Figure S4: RT-PCR analysis of gingival fibroblasts incubated with HSC2 and TR146 necrotic cell lysates overnight in SB431542; Figure S5: RT-PCR analysis of gingival fibroblasts incubated with gingival fibroblast (GF), HSC2, and TR146, and primary oral epithelial cell (POEC) cell lysates overnight; Figure S6: RT-PCR analysis of gingival fibroblasts incubated with HSC2 and TR146 necrotic cell lysates overnight in SB431542; Figure S7: RT-PCR analysis of gingival fibroblasts incubated with recombinant TGF-β overnight; Table S1: The effects of the sonicated cell lysates on STC1, AREG, and C11orf96 expression in gingival fibroblast; Table S2: STC1 immunoassay analysis of gingival fibroblasts in the presence of necrotic cell lysates; Table S3: The effects of the cell lysate of the primary epithelial cell on gingival fibroblast; Table S4: RT-PCR analysis of gingival fibroblasts incubated with HSC2 and TR146 necrotic cell lysates overnight in SB431542; Table S5: RT-PCR analysis of gingival fibroblasts incubated with gingival fibroblast (GF), HSC2, TR146, and primary oral epithelial cell (POEC) lysates overnight; Table S6: RT-PCR analysis of gingival fibroblasts incubated with HSC2 and TR146 necrotic cell lysates overnight in SB431542; Table S7: RT-PCR analysis of gingival fibroblasts incubated with recombinant TGF-β overnight.
