# Supplementary material for: In Vitro Bioassay for Damage-Associated Molecular Patterns Arising from Injured Oral Cells
Source: Bioengineering (Basel). 2024 Jul 5;11(7):687. doi: 10.3390/bioengineering11070687 (PMC11273541; doi:10.3390/bioengineering11070687)
Supplement: Supplementary file 1 [file bioengineering-11-00687-s001.zip › bioengineering-3057195-supplementary.pdf]

bioengineering

MDPI

*Article*

# In Vitro Bioassay for Damage-Associated Molecular Patterns Arising from Injured Oral Cells

**Supplement Figures S1-S7 showing mean and SD of the original Figures in the main document and the respective numbers as Supplement Tables S1-S7**

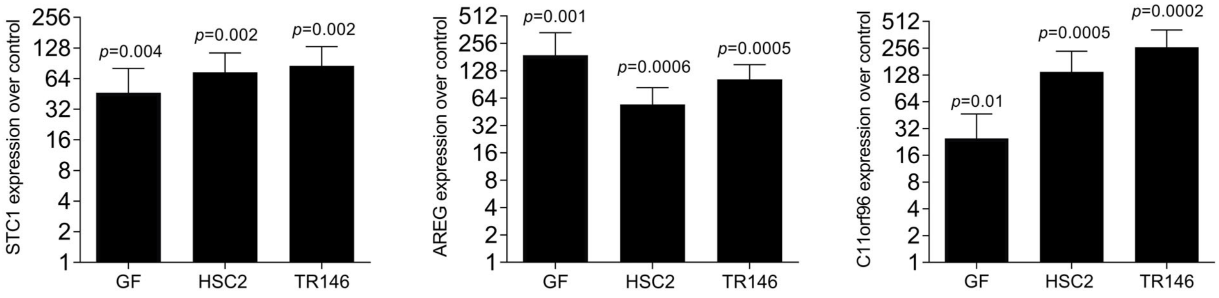


Supplement Figure S1. RT-PCR analysis of gingival fibroblasts in the presence of necrotic cell lysate of gingival fibroblasts, HSC2, and TR146. Data were normalized to untreated control cells with x-fold changes compared to the untreated cells. The analysis was based on a ratio paired t-test.

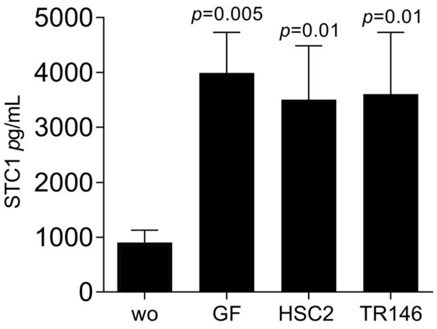


Supplement Figure S2. Immunoassay analysis of gingival fibroblasts in the presence of necrotic cells. Gingival fibroblasts were incubated with necrotic cell lysate overnight, and the immunoassay showed an increase in STC1. Data points represent four independent experiments. The analysis was based on a ratio paired t-test.

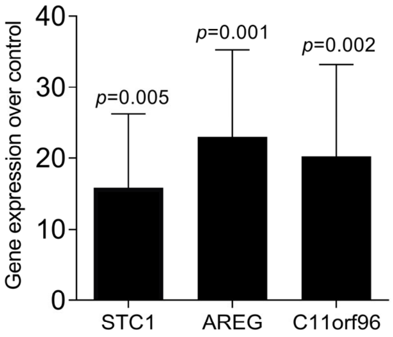


**Supplement Figure S3**. RT-PCR analysis of gingival fibroblasts in necrotic cell lysate of primary epithelial cell lysate. Gingival fibroblasts incubated with necrotic cell lysates overnight, and the gene expression analysis showed an increase in STC1, AREG, and C11orf96 in gingival fibroblasts. Data were normalized to untreated control cells with x-fold changes compared to the untreated cells. The analysis was based on a ratio paired t-test.

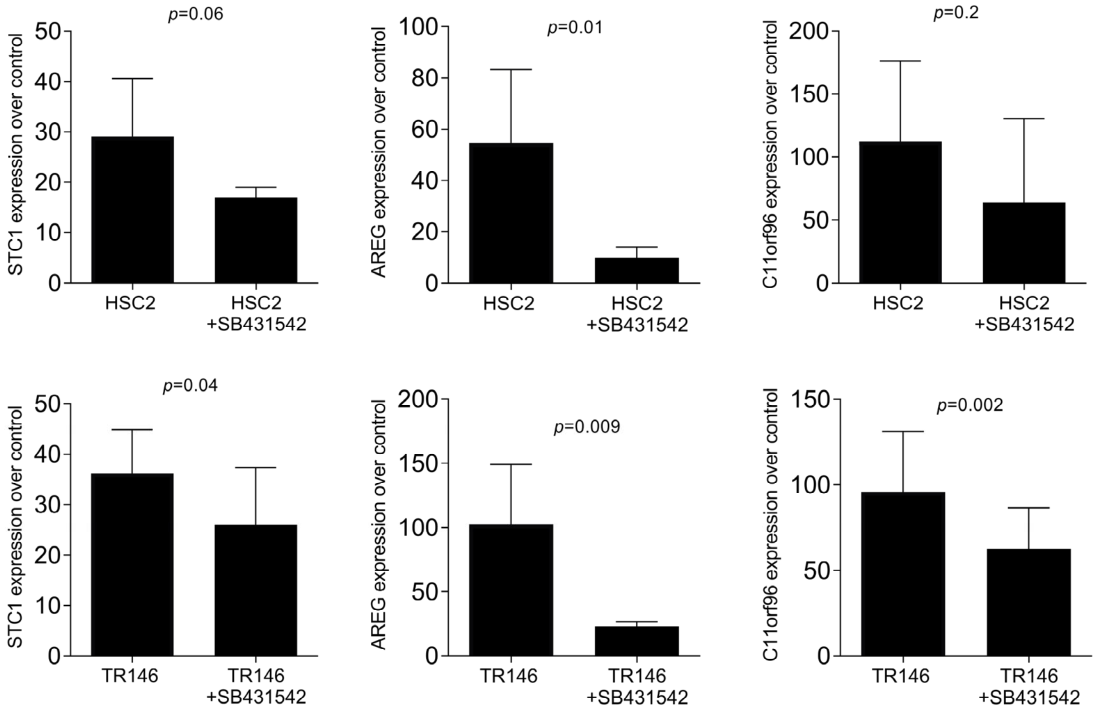


**Supplement Figure S4**. RT-PCR analysis of gingival fibroblasts incubated with HSC2 and TR146 necrotic cell lysates overnight in SB431542. RT-PCR analysis of gingival fibroblasts incubated with necrotic cell lysates with and without the TGF-β RI kinase inhibitor SB431542. Expression analysis showed that blocking TGF-β signalling reduced necrotic cell lysate-induced STC1, AREG, and C11orf96 expression in gingival fibroblasts. Data were normalized against untreated control cells with x-fold changes compared to the untreated cells. The analysis was based on a ratio paired t-test.

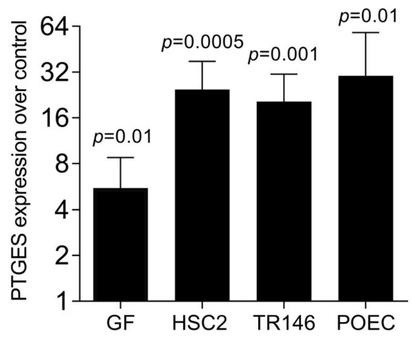


**Supplement Figure S5**. RT-PCR analysis of gingival fibroblasts incubated with gingival fibroblast (GF), HSC2, and TR146, and primary oral epithelial cell (POEC) cell lysates overnight. Gene expression analysis showed an increase in PTGES. Data were normalized to untreated control cells with x-fold changes compared to the untreated cells. The analysis was based on a ratio paired t-test.

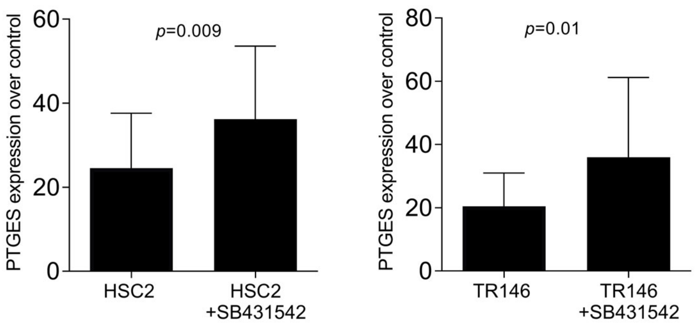


**Supplement Figure S6**. RT-PCR analysis of gingival fibroblasts incubated with HSC2 and TR146 necrotic cell lysates overnight in SB431542. RT-PCR analysis of gingival fibroblasts incubated with necrotic cell lysates with and without the TGF-β RI kinase inhibitor SB431542. Expression analysis showed that blocking TGF-β signalling reduced necrotic cell lysate-induced expression of PTGES in gingival fibroblasts. Data were normalized against untreated control cells with x-fold changes compared to the untreated cells. The analysis was based on a ratio paired t-test.

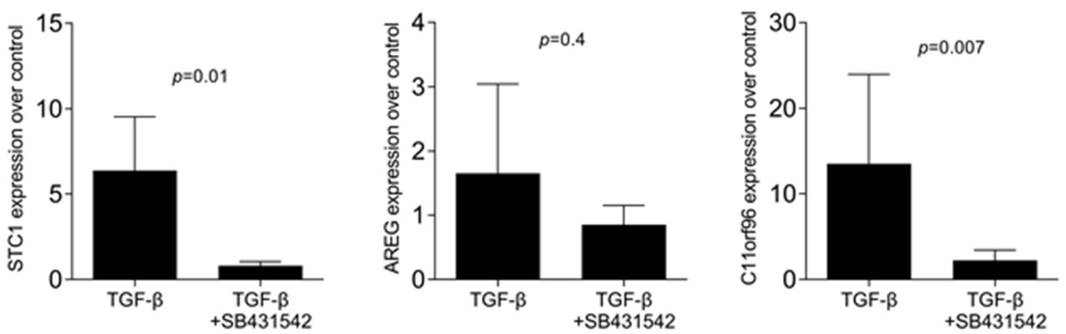


**Supplement Figure S7.** RT-PCR analysis of gingival fibroblasts incubated with recombinant TGF-β overnight. RT-PCR analysis of gingival fibroblasts incubated with recombinant TGF-β with and without the TGF-β RI kinase inhibitor SB431542. Expression analysis showed that blocking TGF-β signalling reduced induced expression in gingival fibroblasts.

Data were normalized against untreated control cells with x-fold changes compared to the untreated cells. The analysis was based on a ratio paired t-test.

Supplement Tables corresponding to Supplement Figures S1-S7

| | STC1 mean ± SD | AREG mean ± SD | C11orf96 mean ± SD |
|---|---|---|---|
| GF lysate | 46.45 ± 29.45 | 189.75 ± 126.83 | 24.75 ± 19.00 |
| HSC2 lysate | 73.73 ± 35.33 | 54.50 ± 24.92 | 138.75 ± 85.21 |
| TR146 lysate | 85.55 ± 40.18 | 102.50 ± 40.31 | 262.75 ± 127.60 |

**Supplement Table S1**. The effects of the sonicated cell lysates on STC1, AREG, and C11orf96 expression in gingival fibroblast. Gingival fibroblasts were exposed to sonicated cell lysates from gingival fibroblasts (GF), HSC2, and TR146 (TR) cells. The x-fold change expression of STC1, AREG, and C11orf96 were normalized to an unstimulated control. The table shows the means and standard deviation (SD) of four independent experiments

| | STC1 mean ± SD |
|---|---|
| wo | 906.50± 192.69 |
| GF lysate | 3990.25 ± 642.20 |
| HSC2 lysate | 3506.00 ± 848.65 |
| TR146 lysate | 3603.00 ± 975.75 |

**Supplement Table S2**. STC1 immunoassay analysis of gingival fibroblasts in the presence of necrotic cell lysates. Gingival fibroblasts were incubated with necrotic cell lysate from gingival fibroblasts (GF), HSC2, and TR146 (TR) cells overnight, and the immunoassay showed an increase in STC1. Data in the table represent the means and standard deviation (SD) of four independent experiments

| | STC1 mean ± SD | AREG mean ± SD | C11orf96 mean ± SD |
|---|---|---|---|
| POEC lysate | 15.83 ± 9.03 | 23.00 ± 10.61 | 20.25 ± 11.19 |

**Supplement Table S3**. The effects of the cell lysate of the primary epithelial cell on gingival fibroblast. Gingival fibroblasts incubated with necrotic cell lysates of primary epithelial cells overnight, and the gene expression analysis showed an increase in STC1, AREG, and C11orf96 in gingival fibroblasts. The x-fold change expression of STC1, AREG, and C11orf96 were normalized to an unstimulated control. The table shows the means and standard deviation (SD) of four independent experiments

| | STC1 mean ± SD | AREG mean ± SD | C11orf96 mean ± SD |
|---|---|---|---|
| HSC2 lysate | 29.05 ± 9.99 | 54.50 ± 24.92 | 112.00 ± 55.65 |
| HSC2 lysate + SB431542 | 17.00 ± 1.75 | 9.90 ± 3.62 | 63.75 ± 57.88 |
| TR146 lysate | 36.15 ± 7.54 | 102.50 ± 40.31 | 95.85 ± 30.79 |
| TR146 lysate + SB431542 | 26.00 ± 9.79 | 23.00 ± 3.08 | 62.75 ± 20.77 |

**Supplement Table S4.** RT-PCR analysis of gingival fibroblasts incubated with HSC2 and TR146 necrotic cell lysates overnight in SB431542. RT-PCR analysis of gingival fibroblasts incubated with the necrotic cell lysates of HSC2 and TR146 with and without the TGF-β RI kinase inhibitor SB431542. Expression analysis showed that blocking TGF-β reduced necrotic cell lysate-induced STC1, AREG, and C11orf96 expression in gingival fibroblasts. The x-fold change expression of

STC1, AREG, and C11orf96 were normalized to an unstimulated control. The table shows the means and standard deviation (SD) of four independent experiments.

| | PTGES mean ± SD |
|---|---|
| GF lysate | 5.55 ± 2.78 |
| HSC2 lysate | 24.55 ± 11.29 |
| TR146 lysate | 20.48 ± 9.09 |
| POEC lysate | 30.25 ± 24.28 |

**Supplement Table S5**. RT-PCR analysis of gingival fibroblasts incubated with gingival fibroblast (GF), HSC2, TR146, and primary oral epithelial cell (POEC) lysates overnight. Gene expression analysis showed an increase in PTGES. The x-fold change expressions were normalized to an unstimulated control. The table shows the means and standard deviation (SD) of four independent experiments

| | PTGES mean ± SD |
|---|---|
| HSC2 lysate | 24.55 ± 11.29 |
| HSC2 lysate + SB431542 | 36.20 ± 15.07 |
| TR146 lysate | 20.48 ± 9.09 |
| TR146 lysate + SB431542 | 35.98 ± 21.87 |

**Supplement Table S6**. RT-PCR analysis of gingival fibroblasts incubated with HSC2 and TR146 necrotic cell lysates overnight in SB431542. RT-PCR analysis of gingival fibroblasts incubated with the necrotic cell lysates of HSC2 and TR146 with and without the TGF-β RI kinase inhibitor SB431542. Expression analysis showed that blocking TGF-β reduced necrotic cell lysate-induced PTGES expression in gingival fibroblasts. The x-fold change expression of STC1, AREG, and C11orf96 were normalized to an unstimulated control. The table shows the means and standard deviation (SD) of four independent experiments.

| | STC1 mean ± SD | AREG mean ± SD | C11orf96 mean ± SD |
|---|---|---|---|
| TGF-β | 6.38 ± 2.74 | 1.65 ± 0.85 | 13.55 ± 9.03 |
| TGF-β+SB431542 | 0.08 ± 0.21 | 1.21 ± 0.26 | 2.23 ± 1.04 |

**Supplement Table S7.** RT-PCR analysis of gingival fibroblasts incubated with recombinant TGF-β overnight. RT-PCR analysis of gingival fibroblasts incubated with the recombinant TGF-β with and without the TGF-β RI kinase inhibitor SB431542. Expression analysis showed that blocking TGF-β signalling reduced induced expression in gingival fibroblasts. The table shows the means and standard deviation (SD) of four independent experiments.

**Supplement Figure S8: Melting of the PCR product at STC1 84°C, AREG 80°C, C11or96 85°C, PTGES 87°C, and GAPDH 82°C and primer sequence.**

| Primers | Sequence Forward | Sequence Reverse |
|---|---|---|
| STC1 | GCAGGAAGAGTGCTACAGCAAG | CATTCCAGCAGGCTTCGGACAA |
| AREG | GTGGTGCTGTCGCTCTTGATA | CCCCAGAAAATGGTTCACGCT |
| C11orf96 | TCACGCCAACACTCTCGCTGAA | CAATCCTCCAGACGCAGTAGCA |
| PTGES | GAGGATGCCCTGAGACACGGA | CCAGAAAGGAGTAGACGAAGCC |
| GAPDH | AAGCCACATCGCTCAGACAC | GCCCAATACGACCAAATCC |

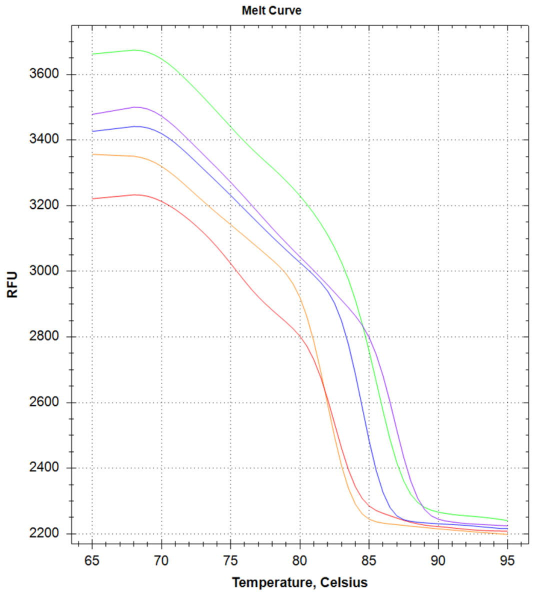


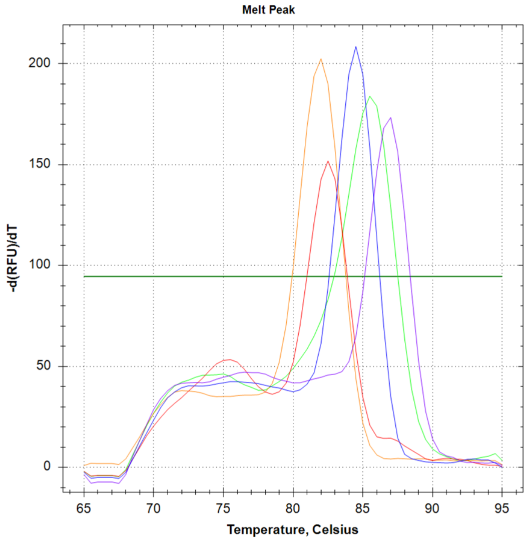


STC1 = blue; AREG = red; C11orf96 = green; PTGES = purple; Gapdh = orange.
